# The effects of far-red light on medicinal Cannabis

**DOI:** 10.1038/s41598-025-99771-6

**Published:** 2025-05-20

**Authors:** Tyson J. Peterswald, Jos C. Mieog, Tobias Kretzschmar, Sarah J. Purdy

**Affiliations:** 1 New South Wales Department of Primary Industries and Regional Development, 105 Prince Street, Orange, NSW 2800 Australia; 2 Southern Cross Plant Science, Southern Cross University, Military Rd, East Lismore, NSW 2480 Australia

**Keywords:** Biochemistry, Physiology, Plant sciences

## Abstract

Far-red (FR) light elicits two distinct processes in plants. First, a shade avoidance response which is triggered when the ratio of red to FR (R: FR) declines. Second, it interacts synergistically with higher frequency wavelengths of light (e.g. red or white) which improves the efficiency of photosynthesis. We investigated whether we could harness these phenomena in medicinal Cannabis to improve yields so that the duration of the photoperiod could be reduced to 10 h (“10L”) whilst returning similar or improved yields compared to a 12 h photoperiod (“12L”). The THC concentrations were elevated in both high THC varieties by the different FR treatments. In Hindu Kush the concentration of THC was elevated by the addition of 4 h of total FR (“10L_2_2D”), and in Northern Lights total cannabinoid yields were increased by nearly 70% over the 12 L control (0.43 versus 0.25 g Plant^− 1^) by the addition of 2 h of FR in darkness after 10 h of light (“10L_2D”). Our results show a strong yield and quality advantage in high THC lines treated with end-of-day FR treatments. Furthermore, a lighting schedule of 10L_2D instead of 12 L would result in a saving of 5.5% in power usage and resultant emissions.

## Introduction

*Cannabis Sativa* is a globally significant plant species with a wide range of uses, including the production of fibre for clothing, seeds for animal and human nutrition and compounds with psychoactive properties for medicinal, religious, and recreational purposes. Currently, the primary target cannabinoids in medicinal cannabis are tetrahydrocannabinolic acid (THCA) and cannabidiolic acid (CBDA)^1^. Through decarboxylation, which converts the acid forms into their neutral forms, THCA becomes Δ^9^-tetrahydrocannabinol (“d9-THC”), and CBDA becomes cannabidiol (CBD). These neutral forms have the ability to interact with the mammalian endocannabinoid system, offering therapeutic benefits for non-communicable illnesses such as sleep disorders, multiple sclerosis, appetite stimulation, and epilepsy^2–4^.

Growing medicinal cannabis indoors and in a glasshouse offer advantages such as climate control and security and precise control over environmental factors such as light, temperature, humidity and CO_2_ levels, allowing growers to create optimal growth conditions for year-round production of maximised yields^5^. Moreover, indoor cultivation provides protection from external abiotic and biotic stresses such as climate, pests and diseases, resulting in more consistent and reliable product quality^5,6^. Light emitting diode (LED) lighting is the preferred choice for indoor cultivation due to its energy efficiency, customisable light spectrum, and low heat output^6^. LEDs can be adjusted to match specific growth stages, promoting healthier plants and maximising yields.

Photosynthesis, the process by which plants convert light energy into carbon-based matter, is influenced by the overall light quality^7,8^. Photosynthetically active radiation (PAR), which ranges from 400 nm to 700 nm and far-red (FR) light wavelengths in the range of 700 nm to 800 nm play a crucial role in plant photosynthetic capacity and crop yields^7^.

Far-red light has been a subject of interest for various plant industries, including medical Cannabis (MC). Plants have evolved to sense FR and react to it as a means to respond to intra and interspecific competition for light. The most recognised response of plants to FR light is the shade avoidance response^9,10^. When plants are shaded by neighbouring vegetation, the chlorophylls of the rival plants selectively absorb the red and blue wavelengths, whilst reflecting or transmitting the FR. This causes a decrease in the red light to far red (R: FR) ratio which triggers a range of responses, including stem elongation, leaf expansion, and altered flowering patterns which are part of shade avoidance mechanisms^11^. The response to FR light is mediated by phytochromes, photoreceptors that sense different wavelengths of light^12^. The change in proportions of R: FR cause a concurrent shift in the proportions of the active phytochrome form, Pfr, to the inactive form, Pr^13^. The inactive forms prevent the breakdown of a group of transcription factors called PHYTOCHROME INTERACTING FACTORS (PIFs), instead allowing them to relocate from the nucleus to the cytoplasm. There, they initiate the responses frequently associated with shade avoidance, by activating genes involved in auxin biosynthesis and cell elongation^14^.

In experiments using monochromatic light, photosynthesis rapidly declines as the wavelength of light increases towards the FR end of the spectrum; this was described as the “red drop” by Emerson and Lewis in 1943^15^. However, when monochromatic FR light was supplemented with lower wavelength red light, an interaction was observed that resulted in a photosynthetic rate that was greater than when either spectrum was applied alone. This synergistic effect was termed the Emerson effect^16^. In more recent years a series of studies^8,17–20^ have also shown that a synergistic effect occurs when certain spectra such as red-blue or warm white, are supplemented with FR^18,20^. The explanation for this phenomenon lies in the fact that photosystems I and II, which operate in series for the electron transport chain, need to be excited approximately equally for optimal photosynthetic efficiency^21^ and this is not always the case, especially under artificial lighting. Zhen and van Iersal (2017)^20^ demonstrated that the chlorophyll fluorescence declined (a measure of greater efficiency of electron transfer from PSII to PSI) following the application of FR under a red/blue spectrum, and the authors concluded that this was a result of FR preferentially exciting PSI allowing more rapid re-oxidation of plastoquinone^22,23^. The re-oxidised plastoquinone can then accept more electrons from PSII chlorophylls allowing the PSII reaction centres to re-open more quickly, thus improving the rate and efficiency of photosynthesis^20,23^. Thus, their work showed that far-red light can increase the photosynthetic efficiency of shorter wavelength light that over-excites PSII.

Therefore, the addition of FR light radiation in the spectrum can both modify plant morphology through shade avoidance and enhance the efficiency of photosynthesis. Understanding and harnessing the effects of FR light could have significant implications for optimising medical cannabis cultivation and improving crop quality.

Previous experiments conducted in various crops have demonstrated multiple outcomes when applying FR light that could be beneficial in Cannabis production systems. For example, in *Glycine max* (soybean) it was observed that under a low R: FR ratio, there was an increase in photo-assimilate content, indicating enhanced photosynthetic activity, and a rise in sucrose and starch content, suggesting potential metabolic shifts^9^. The addition of FR light to the light spectrum has been documented to hasten the flowering process across various plant species, such as lentil^24^. It is important to note, however, that lentil is a long-day flowering plant. In short-day flowering plants, recent experimentation has demonstrated that the addition of FR light during a 12-hour period of darkness significantly delayed the onset flowering in Cannabis and Glycine max (Soybean)^9^. These results highlight the significant impact that the R: FR ratio can have on plant development and metabolic processes in various crop species.

Flowering in most varieties of Cannabis is strongly regulated by photoperiod. In the Cannabis industry, an inductive flowering photoperiod of 12 h light: 12 h dark (12 L:12D) is enforced by black-out curtains and supplementary lighting. It has previously been shown that extending the flowering photoperiod from 12 h light to 14 h light was detrimental to the cannabinoid contents in the high THC varieties, Hindu Kush and Northern Lights, whereas reducing the photoperiod to 10 h caused a flower biomass decrease reducing the yield^25^. Therefore, in these two lines the industry standard 12 L:12D was optimal for production. However, if the addition of FR light could increase the rate of photosynthesis and/or initiate metabolic shifts, a shorter photoperiod could potentially be employed which would result in lower energy costs and carbon emissions. This is an enticing prospect as the economic cost of lighting in Cannabis cultivation constitutes a significant portion of overall operational expenses. It estimated to require Australian growers 4kWh of energy to produce 1 g of dried flower per m^2^^26^, therefore, indoor cultivation using 100% LED lighting for 12 or 18 + hours a day (for flowering or mother generation, respectively) results in considerable electricity costs. High CO_2_ emissions go hand-in-hand with high electricity usage and recent modelling from the US reported that 1,283-5,184 kg CO_2 e_ per kg of dried flower was emitted from indoor grow facilities, with 238–805 kg CO_2 e_ of that value being derived from grow lights^5^.

This study investigated whether hours of full-spectrum light could be substituted by increased exposure to FR. To determine this, we tested the effects of supplementary FR, either at the end of the light period, the start of the dark period, or both, on plant height, flowering, biomass yield and cannabinoid contents under either a control (12 L) or shortened photoperiod (10 L).

Under natural conditions as the sun sets, the amount of far red light exceeds the amount of red light^27^ and previous studies in other species have shown stronger responses to FR treatments applied at the end of day rather than earlier timepoints. For these reasons, we chose to apply the FR treatments at the end of the photoperiod in our indoor system.

## Results

The primary aim of this study was to investigate whether the addition of end-of-day FR could supplement for 2 h of full spectrum light. The 6 treatments used to test this were:

10 L = 10 h photoperiod, no FR.

10L_2 = 2 h of FR in the last 2 h of the light period (hours 8–10).

10L_2D = 2 h of FR applied at the start of the dark period (hours 10–12).

10L_2_2D = 4 h of FR applied as 2 h in the light and 2 h at the start of the dark (hours 8–12).

12 L = the control 12 h photoperiod (no FR).

12L_2_2D = 4 h of FR applied as 2 h in the light and 2 h at the start of the dark (hours 10–14).

### Plant height

**Fig. 1:**
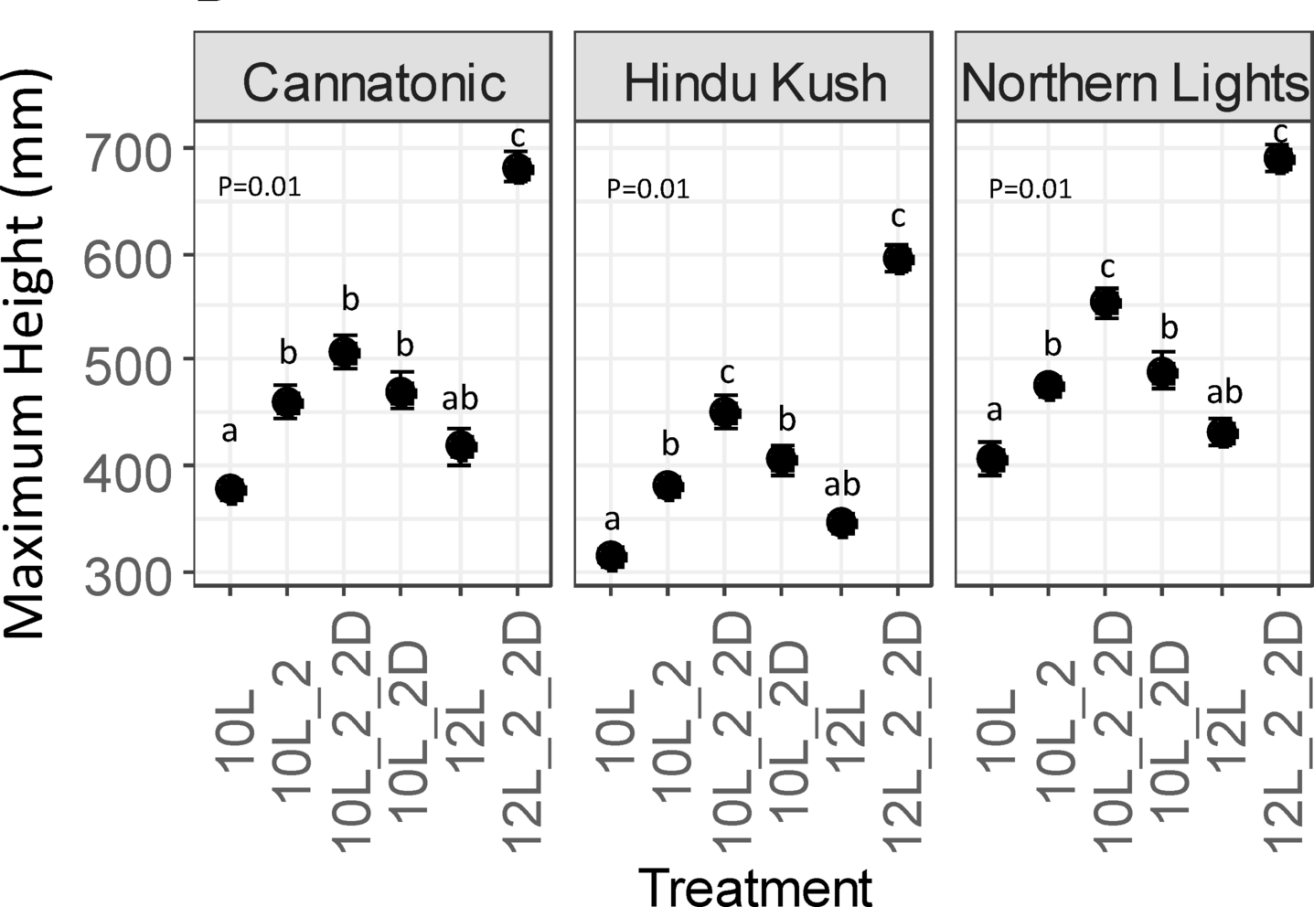
Final plant height. *N* = 7. Results of an ANOVA and Tukey HSD are shown (P = < 0.05). Treatment abbreviations *10 L* 10 h photoperiod, no FR, *10L_2* 2 h of FR in the last 2 h of the light period, *10L_2D* 2 h of FR applied at the start of the dark period, *10L_2_2D* 4 h of FR applied as 2 h in the light and 2 h at the start of the dark, *12 L* 12 h photoperiod (no FR), *12L_2_2D* 4 h of FR applied as 2 h in the light and 2 h at the start of the dark.

Plant height was increased by the application of FR in all genotypes as all 10 L treatments with FR were significantly taller than 10 L (no FR) (Fig. 1). The 12 L (no FR) treatment resulted in significantly shorter plants compared to the 10L_2_2D and the 12L_2_2D treatment resulted in significantly taller plants than all other treatments.

### Plant biomass

**Fig. 2:**
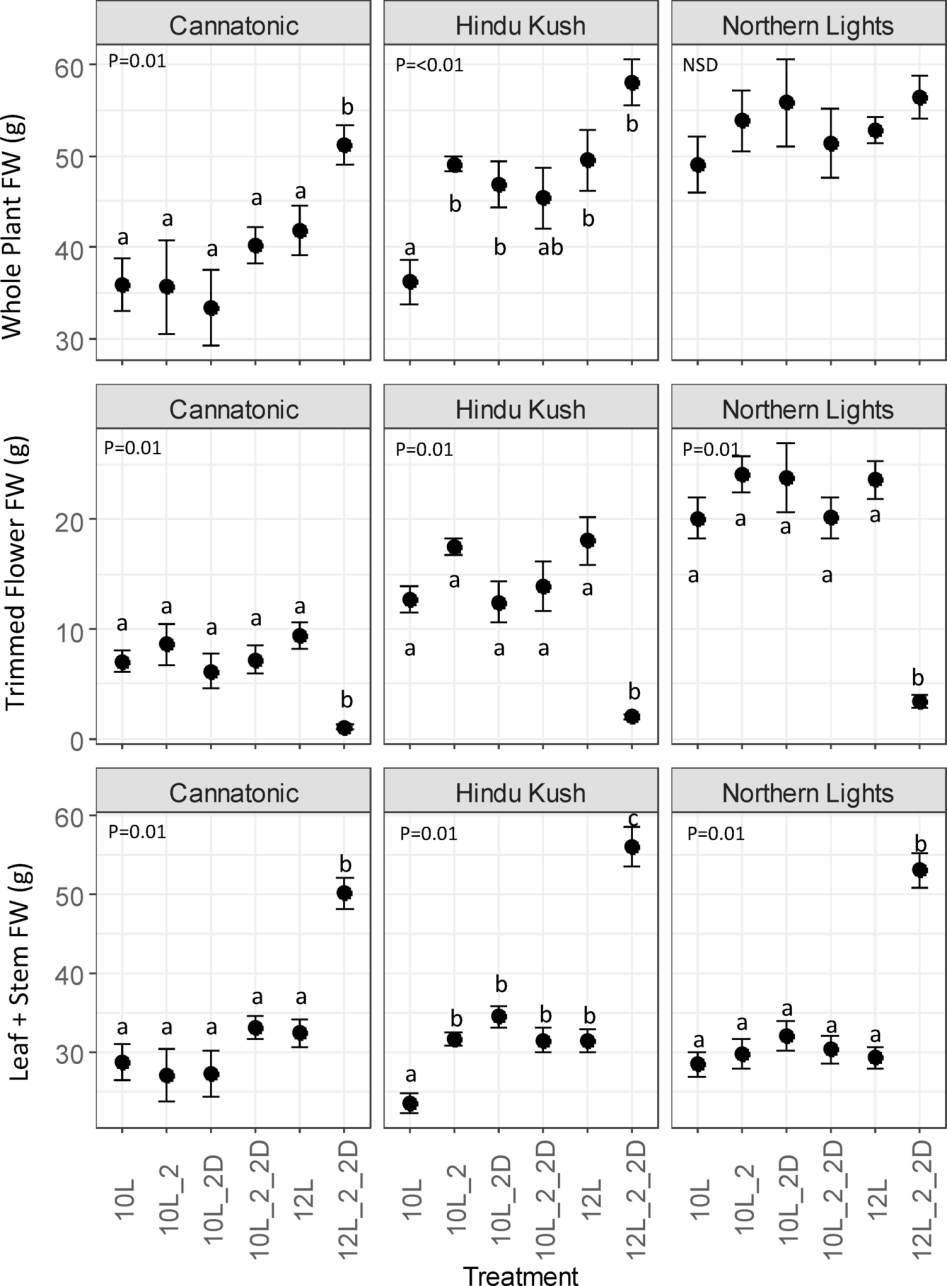
Plant biomass (g FW). *N* = 7. Results of an ANOVA are shown with a Tukey HSD test (P = < 0.05), NSD = no significant differences. Treatment abbreviations *10 L* 10 h photoperiod, no FR, *10L_2 = 2* hours of FR in the last 2 h of the light period, *10L_2D* 2 h of FR applied at the start of the dark period, *10L_2_2D* 4 h of FR applied as 2 h in the light and 2 h at the start of the dark, *12 L* 12 h photoperiod (no FR), *12L_2_2D* 4 h of FR applied as 2 h in the light and 2 h at the start of the dark.

Whole plant fresh weight (FW) biomass was significantly increased in Cannatonic and Hindu Kush in the 12L_2_2D treatment compared to the control (12 L), wheras no significant differences between treatments were observed for Northern Lights (Fig. 2). In Hindu Kush, the 10 L treatment also resulted in a significantly lower whole plant biomass compared to all treatments, except 10L_2_2D.

The trimmed flower weight was significantly reduced by the 12_2_2D treatment in all genotypes, whereas the leaf and stem (vegetative) biomass showed the opposite effect, being increased by the 12L_2_2D treatment in all genotypes.

The leaf + stem FW biomass of Hindu Kush was significantly reduced in 10 L compared to all other treatments.

### Cannabinoid concentration

The cannabinoid data for 12L_2_2D was not quantified as the plants did not produce enough dry flower biomass yield for cannabinoid analysis.

**Fig. 3:**
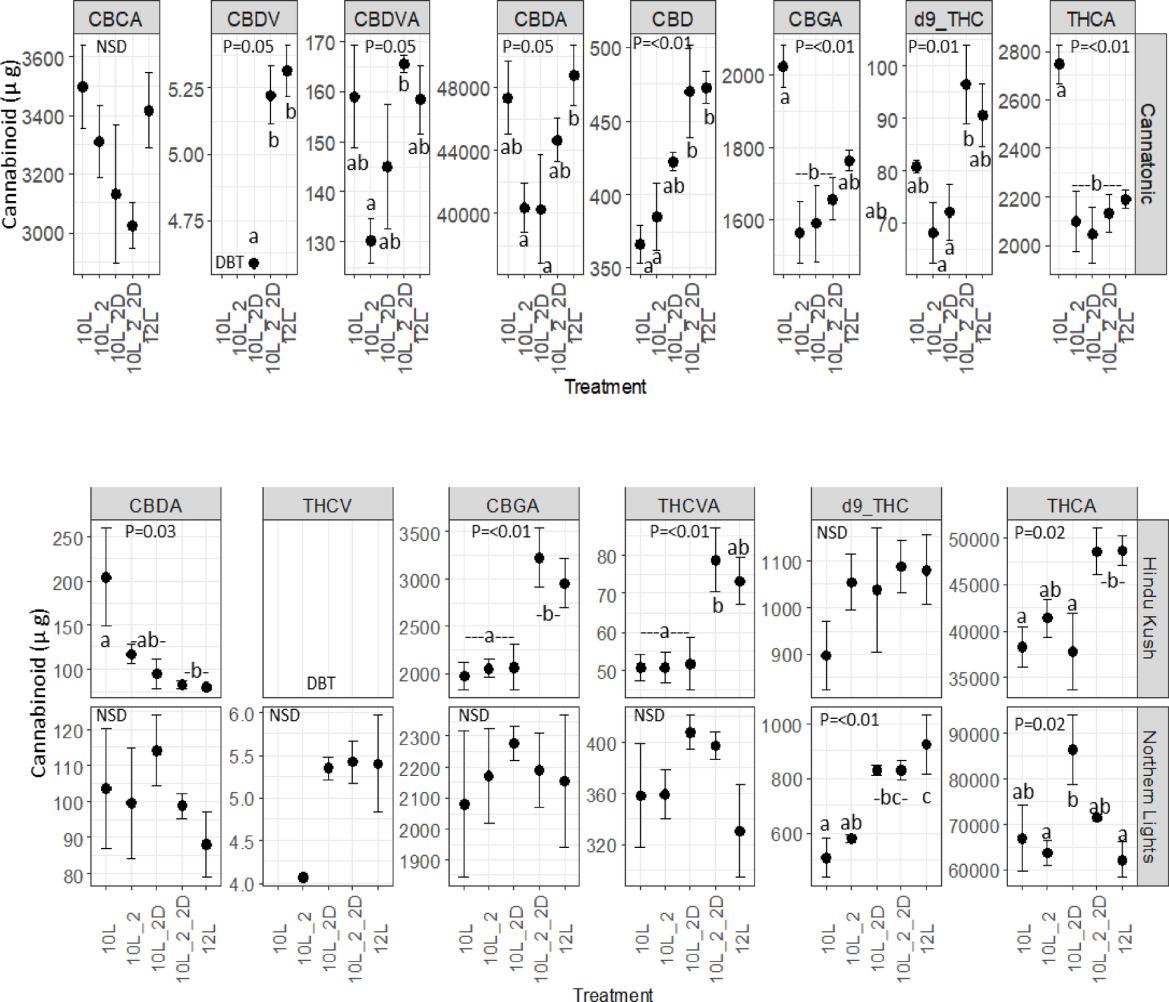
Cannabinoid concentrations µg g DW. *N* = 4 +/-SE. The results of an ANOVA and Tukey HSD test are shown (P = < 0.05), NSD = No significant difference. Treatment abbreviations *10 L* 10 h photoperiod, no FR, *10L_2* 2 h of FR in the last 2 h of the light period, *10L_2D* 2 h of FR applied at the start of the dark period, *10L_2_2D* 4 h of FR applied as 2 h in the light and 2 h at the start of the dark, *12 L* 12 h photoperiod (no FR).

Seventeen cannabinoids were analysed^28^ but five were either not dectected in any sample (CBN, Δ^8^-THC, CBLA, CBL) or detected in some, but below the threshold for quantification (CBNA). Different cannabinoids were detected in the three genotypes. In Cannatonic, eight cannabinoids were detected, whereas in the high THCA accumulating lines, Hindu Kush and Northern Lights, six Cannabinoids were detected, respectively (Fig. 3).

In Cannatonic, seven out of the eight quantified cannabinoids showed significant differences in concentration in response to treatment (Fig. 3). The neutral cannabinoids, CBDV, CBD and d9-THC all showed a similar response where the abundance was highest equally in 10L_2_2D and 12 L and lower in the other 10 L treatments. The 10L_2D treatment resulted in a higher concentration of CBD than either 10 L or 10L_2 and produced a low but detectable level of CBDV, where as 10 L and 10L_2, did not. The concentration of THCA and CBGA was significantly higher in 10 L compared to all other treatments. For the highest abundant cannabinoid in Cannatonic, CBDA, the treatments containing FR all appeared to repress accumulation.

The same cannabiniods were detected in both Hindu Kush and Northern Lights but THCV was below the level of quantification in Hindu Kush (Fig. 3). Differences in response to treatment were observed between these two high THC lines. In Hindu Kush, the acidic cannabinoids CBGA, THCVA and THCA were equally high in 10L_2_2D and 12 L but lower in the other treatments, and CBDA was highest in 10 L but lower in all other treatments. In Northern Lights, significant differences in response to treatment were only observed in d9-THC and THCA. The concentration of d9-THC was significantly higher in 10L_2D, 10L_2_2D and 12 L compared to the 10 L and 10L_2 treatments. THCA was significantly elevated by ~ 25% in the 10L_2D treatment compared to either 10–12 L. THCV was only observed in the 10L_2D, 10L_2_2D and 12 L treatments, it was also observed in 10L_2 but only in a single sample (Fig. 3). No significant differences in the concentration of THCVA was observed for Northern Lights, but the concentrations trended higher in 10L_2D and 10L_2_2D compared to the other treatments.

### Total yield of target cannabinoid

**Fig. 4:**
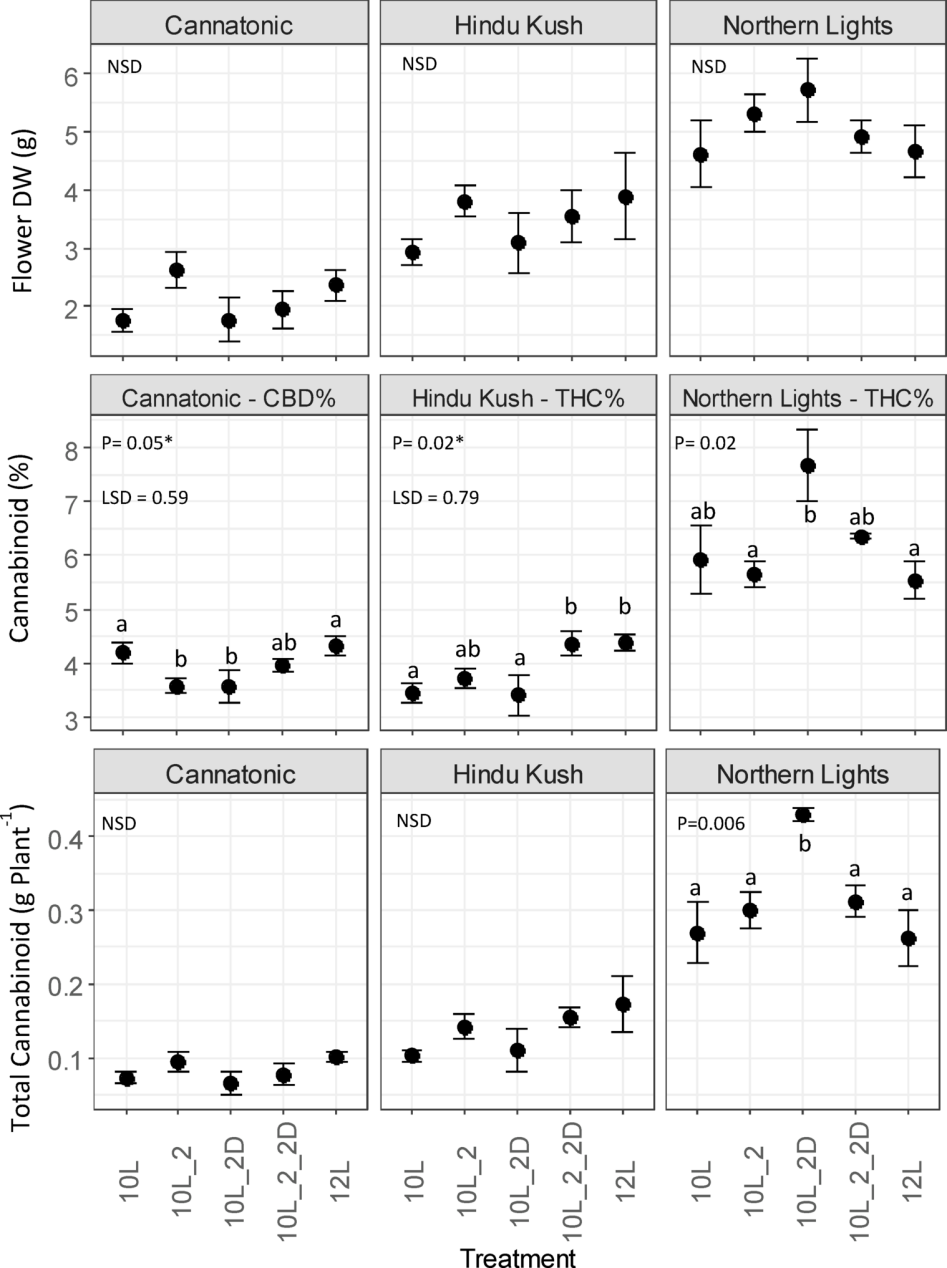
Effect of treatment on total cannabinoid yield (CBD – Cannatonic, THC – Hindu Kush and Northern Lights). *N* = 4, +/-SE. Results of an ANOVA and Tukey HSD test are shown. In ANOVA values marked with and asterisks*, a LSD test was used in place of Tukey P = < 0.05. NSD = No Significant Difference. Treatment abbreviations *10 L* 10 h photoperiod, no FR, *10L_2* 2 h of FR in the last 2 h of the light period, *10L_2D* 2 h of FR applied at the start of the dark period, *10L_2_2D* 4 h of FR applied as 2 h in the light and 2 h at the start of the dark, *12 L* 12 h photoperiod (no FR).

No significant differences were observed between treatments for dry flower yields (g Plant^-1^) for any of the genotypes (Fig. 4). In contrast, all lines showed a response to treatment in the target cannabinoid % (total CBD or THC – see Materials and Methods for formula). In Cannatonic, treatments with 10L_2 or 10L_2D both showed depressed concentrations of total CBD compared to 10 L and 12 L. In Hindu Kush the total THC% in 12 L and 10L_2_2D were elevated compared to the other treatments whereas in Northern Lights the highest concentration was in the 10L_2D treatment (Fig. 4). No significant differences in total cannabinoid yield (g Plant^-1^) were observed in Cannatonic or Hindu Kush despite the observed differences in target cannabinoid % (Fig. 4). However, in Northern Lights, the combined effect of increased THC% in 10L_2D and the slightly raised (though non-significant) increase in flower biomass in the same treatment resulted in an increase of 70% (0.43 g Plant^-1^) of yield compared to 10 L (0.27 g Plant^-1^) or 12 L (0.25 g Plant^-1^) (Fig. 4).

### Energy use

The energy use, associated costs and projected carbon emissions from the five lighting treatments that resulted in a commercially viable flower yield are shown in Table 1. The different lighting settings resulted in different power demands. The lowest was 10 L and the highest being 12 L. If a grower was to use the 10L_2D treatment that resulted in the 70% yield advantage in Northern Lights, a 5.5% saving on electricity and emissions would be made compared to using the standard 12 L (Table 1). These values reflect only the lighting energy use and not electricity use for the whole facility (which includes, for example, temperature control).

**Table 1:** Energy use and emissions per grow light calculated under the assumption that CO_2_ emissions are set at the sum of scope 2 and scope 3 emissions for NSW of 0.79 kg CO_2_-e /kWh^29^.

Treatment	kWh d^− 1^	kg CO_2−_e *p*.a.
10 L	3.68	967.10
10L_2	3.86	1015.46
10L_2D	3.91	1027.55
10L_2_2D	4.09	1074.85
12 L	4.14	1087.99

## Discussion

All three genotypes showed increased plant height in the treatments that received FR with the tallest plants being observed in 10L_2_2D and 12L_2_2D, most likely because these treatments had the longest daily duration of reduced R: FR ratio (4 h) and therefore induced a stronger shade response. This observation is consistent with findings reported in multiple other species that the addition of FR increases plant height^7,30,31^. It is known that the response of plants to shading is strongest when the shift in R: FR is sensed at dusk^10^. This finding was discovered in Arabidopsis in which the effect on hypocotyl elongation was observed following application of 2 h of a low R: FR at different times of day. When the treatment was received at dusk, hypocotyl elongation increased 25–30%, whereas receiving the signal earlier had a reduced response and at dawn even caused a small but significant decrease in elongation compared to controls^10^. In a further study, shading was applied in 2 h blocks in the lead up to dusk (e.g. hours 6–8, 7–9, 8–10). The authors found that there was no change in response compared to unshaded plants when shade was applied at hours 6–8, but there was doubling in response if shade was applied an hour later at 7–9 (the end of the photoperiod was at hour 10)^32^. If the response described in Arabidopsis is similar for Cannabis, this means that applying the FR treatments at the end of the day as done in this study (i.e. dusk) would have had the strongest promotion of stem elongation. Our results further indicate that the magnitude of response can be manipulated via exposure time. Arabidopsis is a long-day flowering plant and flowering is accelerated by a low R: FR ratio. As with the hypocotyl response, flowering under low R: FR is also regulated by the PHYB phytochrome signalling pathway^33^. In mutants of PHYB, the diurnal transcriptional expression of flowering locus T (FT), is strongly upregulated and consequently flowering occurs two-weeks earlier than wild-type^33^. Whether the flowering response, like the hypocotyl, is also responsive to FR treatments applied at different times of day is unknown, but as the governing regulatory pathways are similar it seems reasonable to assume. We have previously shown that in Hindu Kush, Northern Lights and Cannatonic, flower yields were optimised in a 12–14 L, but reduced in 10 L^25^. These pieces of evidence offer an explanation as to why such an extreme delay in flowering and increase in height was observed in the 12L_2_2D compared to the same treatment 2 h earlier at 10L_2_2D; the response to shading could be more exacerbated in the last hours of the optimum photoperiod for reproductive growth (10–14 h), rather than earlier in the photoperiod (8–12 h).

In Cannatonic and Hindu Kush, an increase in whole plant biomass was observed under 12_2_2D compared to 12 L. This shows that the inclusion of FR can further promote biomass accumulation for some varieties, despite not directly contributing to photoassimilation. The effect was strongest in plants grown under 12_2_2D, possibly due to changes in biomass partitioning towards vegetative matter rather than flowers. In citrus trees, flowering has been shown to be the growth stage with the highest consumption of photoassimilates^34^, demonstrating that more energy is required to produce the same mass of flowers as vegetative biomass. Therefore, the application of FR repressed flowering and allowed repartitioning of reserves into vegetative mass. However, for Hindu Kush, this explanation is not adequate as no reduction in flower biomass was observed with the increase in vegetative growth in any of the 10 L FR treatments compared to 10 L. In this case, it appears that the addition of 2 h of FR promoted vegetative growth and maintained reproductive growth. This may have occurred via the phenomenon reported in a series of papers by Zhen et al.^8,17–20^ in which the balance of excitation between PSI and PSII is improved by the addition of FR into the light spectrum, leading to improved efficiency of photosynthesis. The phenomenon was evident when either sunlight or red/blue LED light was supplied to lettuce plants and then supplemented with 18 narrow bands of FR light to observe the change in the quantum yield of PSII (Φ_PSII_)^19^. The Φ_PSII_ was elevated at all spectrums tested compared to Φ_PSII_ without the addition of the FR^19^. Thus, an improvement in photosynthetic efficiency offers an explanation for the increase in biomass in Hindu Kush when the red/blue/white LED spectrum used for this study was supplemented with FR. However, it is clearly a genotype-specific phenomenon that is not detectable, at least under the treatments we provided, in Cannatonic and Northern Lights.

In Hindu Kush grown at 10 L, we observed increases in the acidic cannabinoid concentrations (except CBDA) in response to 4 h of FR, but smaller, or no change in response to only 2 h (either before or after onset of dark period). This suggests that a dose response exists and that 2 h was inadequate to initiate a change in cannabinoids. Conversely, in Northern Lights the strongest positive effect on THC was observed when FR was applied only in the dark (10L_2D). As application in the dark resulted in a R: FR ratio of 0, because there was only FR, this could suggest that an increase in THCA in Northern Lights only occurs under an extremely low R: FR ratio. An effect of FR on cannabinoids has been previously reported in which the high THC genotype “G-170” was observed to have reduced THC concentrations in a treatment with the lowest R: FR ratio (high pressure sodium)^31^. The authors concluded that this could be either because of the low R: FR ratio, or the absence of blue light in this spectrum^31^. As our plants were not deprived of blue light, our results suggest that a very low R: FR is actually beneficial for THCA accumulation, with the absence of blue light likely causing the detrimental effect observed in the previous study^31^.

In terms of the total cannabinoid yield (g plant^− 1^), Northern Lights responded differently to the FR treatments compared to Hindu Kush and Cannatonic. In Northern Lights, the application of 2 h of FR in the dark after 10 L resulted in an increase in total THC yield (g Plant^-1^), compared to the control 12 L treatment. This was mainly due to a significant increase in total THC % plant^-1^, rather than an increase in biomass yield, which was not significant. This is of interest for growers producing a dried flower, inhalable, product as this treatment produced a crop of higher THC concentration flowers, with two hours less full-spectrum light.

From a crop management perspective our results have several important implications. Many growers are shifting to vertical farming systems which require a set plant height and distance away from the LEDs. Having a high ratio, or excluding FR, will result in more compact plants. In order to not disrupt flowering and maturity, supplementing with FR light needs to be carefully considered, as our findings demonstrate that 12L_2_2D delayed flowering and demolished flower biomass yield. There are numerous studies that have shown flowering acceleration in response to FR application e.g. tomato^35^, petunia and snapdragon^36^. However, these examples are all long-day flowering plants, whereas in short-day flowering, our results and previous reports^37^, suggests that extended application (e.g. 12L_2_2D) of FR it is more likely to delay flowering, which is not desirable for growers. However, lesser amounts of FR application (2 h) in a 10 L photoperiod showed no detrimental effect on flowering. Our study did not address differences in response to varying ratios of R: FR (only duration at a set ratio of 0.43). In a recent study into the effect of full-spectrum light intensity, Cannabis plants were exposed to a linear gradient of intensity which allowed for response curves to be plotted^38^. A similar experiment using FR would be beneficial to allow for the optimum ratio(s) to be identified which could then improve treatment efficiency. There is also scope to optimise the application of a far-red treatment from the overhead lighting used in our system to an intra-canopy lighting system. The use of intra-canopy lighting to partially replace overhead light has been shown to improve yields in some glasshouse crops, at least in part through reduced reflectance^39,40^. If the efficiency of the light application could be improved, this could further reduce energy usage and the respective carbon footprint.

To return to the original question posed in the introduction: can the addition of FR in a 10 h photoperiod substitute for extra hours in full spectrum light? The biggest yield increase was in Northern Lights in 10L_2D, in which the yield was 70% higher compared to the control 12 L. However, as this observation was variety specific, with no changes in cannabinoid yields in Cannatonic and Hindu Kush, a grower would first have to test the effect of this treatment on their specific varieties.

These findings have significant implications for the cannabis industry, specifically for energy consumption, with electricity being a significant cost for cultivation. Consequently, the preference is to achieve productivity improvements without extending, and ideally reducing, the duration of the artificial lighting period. If growers were to utilize a 10L_2D treatment instead of 12 L, a saving of 5.5% would be made on power and carbon emissions. In a report by PharmOut Pty Ltd^26^, it was reported that for a hypothetical Australian indoor facility, 4000–6000 kWh of electricity was required per kg of dried flower produced, and 38% of electricity was used on lighting. If we assume an intermediate value of 5500 kWh /kg, 38% = 2090 kWh /kg. A reduction of 5.5% would equate to a saving of 115 kWh. In NSW, electricity is charged at approximately $0.338 kWh^41^ therefore this would equate to a saving of $39 / kg dried flower and assuming a CO_2_ emission rate of 0.79 kg CO_2_ e/ kWh^29^ a saving of 91 kg CO_2_ e/ kg would be made.

## Conclusions

The application of end of day FR can increase the cannabinoid concentration in high THC varieties of medicinal Cannabis. There was no effect of our FR treatments on flower biomass yields. The cannabinoid responses to FR treatments differed between genotypes and a 4 h dose in combination with a 12 h photoperiod was detrimental for yield in all tested. This evidence shows that, where far red is concerned, you can have too much of a good thing. The increase in Cannabinoid % when FR is applied in a shortened photoperiod allows for the same yields to be produced with a lower energy and carbon footprint.

## Methods

### Plant material and growing conditions

Three medicinal cannabis genotypes provided by Cann Group Ltd (https://www.canngrouplimited.com/, accessed on 22/03/2024) were employed in this study. These genotypes included one high CBDA strain, “Cannatonic,” and two high THCA strains, “Hindu Kush” and “Northern Lights” (previously referred to as “CBD1,” “THC6,” and “THC1,” respectively)^42^. These genotypes had also been previously used in the same growth system in our study on photoperiod responses^25^. These lines were initially chosen because they were all popular medicinal cannabis cultivars but phenotypically and genetically distinct, thus provided information directly relevant to the industry while also providing an indication of the genotype-specificity of observed responses.

All plants were cultivated in a secure facility approved by the Australian Government Department of Health and Aged Care Office of Drug Control (ODC). The experiments were carried out under a Commonwealth license and associated permits. Temperature and humidity were maintained at 25 °C and 50%, respectively. The plants were grown in controlled environments (CE) throughout their life cycle, and they were moved within the CEs on a weekly basis during the flowering period to account for any environmental variations.

The cloning and propagation method used has been previously documented^42^. Experimental plants were cloned from donor mothers. Approximately 15 cm of new growth stems were excised from the mother plants. All leaves on the stem sides were removed, leaving only the top leaf bunch. The bottom of the stem was cut diagonally across a node, creating a clone approximately 12 cm in height. The top leaf bunch was trimmed to the height of the smallest emerging leaf to reduce water loss and prevent overlapping in the propagation dome. The bottom 1 cm of the stem, where roots would form, was lightly scraped with a scalpel, dipped in hormone gel (Clonex Purple, Yates, DuluxGroup, Clayton, Australia), and placed in an organic propagation cube (Eazyplug CT12, Goirle, The Netherlands, eazyplug.nl).

Once the propagation tray was filled with new clones, it was positioned in a propagation dome (Smart Garden heavy-duty 3-piece propagation kit, Epping Hydroponics) for 14 days. The clones were subjected to an 18-hour light/6-hour dark (18 L:6D) photoperiod in a growth cabinet (Conviron A2000, Conviron Asia Pacific Pty Ltd, Grovedale, Australia) at a light intensity of 100 µmol m² s⁻¹ and a temperature of 25 °C. Humidity was gradually reduced over the 14-day propagation period. Plants with established roots were then transplanted into 1.8-litre pots containing a 30:70% blend of perlite and coco-coir with an electrical conductivity (EC) of < 0.5 mS/cm (Professors Nutrients, Australia). Subsequently, the plants were transferred to controlled flowering environments under a photosynthetically active radiation (PAR) of 600 µmol m² s⁻¹ (Heliospectre Grow Light, LX602C) and maintained under an 18 L:6D photoperiod for an additional 5 days as a hardening period to acclimate to the new environment. After the additional 5 days, the photoperiod was switched to one of the 6 treatments described below and flowering was initiated. The temperature ranged from 20 ^o^C (night) to 26 °C (day), and blackout curtains prevented light leakage. The plants were watered and fed using a commercial fertigation recipe with an electrical conductivity (EC) of 2.2 mS/cm and a pH of 6. As there was an odd number of plants (21) within each treatment, plants were spaced 10 cm apart in 40 × 50 cm grid with an extra plant on the end of the row. As plants were kept small (see Peterswald et al. 2023), this setup assured that plants did not shade each other. To avoid edge effects plant positions were shifted in the chambers twice per week during watering.

### Treatments

Each treatment contained 7 replicates of each of the 3 genotypes in a fully randomised design. The six zones were programmed to one of each of the following photoperiods with different timing applications of FR as presented in Table 2. All plants were maintained in these treatments for 40 days, which was the day after cloning (DAC) 70, which is the end point of the flowering treatments.

The 6 programmed zones were controlled using Heliospectre Software (heliospectra.com). A description of each six light spectrum treatments is described in Table 3. The light intensity drops for all treatments from hour 8 because the maximum power (µMol m^2^ S^-1^) of the red light band (660 nm) is more powerful than the maximum of the FR band (735 nm) therefore, in order to achieve a low ratio, the FR had to be turned up and the red band down. Treatments not receiving FR in the light period were treated the same to ensure that the daily PAR was consistent between treatments. The height of the plants from the lights was not periodically adjusted because our experiments were designed to represent conditions in commercial facilities in which the bench height and lighting infrastructures are typically fixed. The temperature within the treatments at canopy height was 26 ^o^C under the full spectrum lights and 24 ^o^C under FR only and 20 ^o^C with all lights off.

**Table 2:** Six treatments applied. Seven replicates per genotype were included for each treatment.

Treatment 1 (Light/Dark)	Total FR hours	Name	Description
10 L:14D	0	10 L	Stayed under 10 L:14D for the duration of the experiment
10 L:14D (FR 8–10)	2	10L_2	Applied FR light at photoperiod hours 8–10
10 L:14D (FR 10–12)	2	10L_2D	Applied FR light at photoperiod hours 10–12 (in the dark)
10 L;14D (FR 8–12)	4	10L_2_2D	Applied FR light at photoperiod hours 8–12 (2 h in the light 2 h in dark)
12 L:12D	0	12 L	Stayed under 12 L:12D for the duration of the experiment
12 L:12D (FR 10–14)	4	12L_2_2D	Applied FR light at photoperiod hours 10–14 (2 h in the light 2 h in dark)

**Table 3:** The PAR, % of each colour in the total spectrum and ratios for the treatments.

µMol m^2^ S^− 1^								
Treatment	PAR	Hour	B 450 nm	*R* 660 nm	FR 735 nm	W 5700 K	*R*/FR	Daily PFD Mol m^2^ S^− 1^
10 L	854	0–8	100.00	474.00	0.55	280.00	861.82	27.52
	404	8–10	100.00	23.70	0.55	280.00	43.09	
	0	10–12	0.00	0.00	0.00	0.00	-	
	854	0–8	100.00	474.00	0.55	280.00	861.82	27.91
10 L + 2 L	404	8–10	100.00	23.70	55.00	280.00	0.43	
	0	10–12	0.00	0.00	0.00	0.00	-	
	854	0–8	100.00	474.00	0.55	280.00	861.82	27.52
10 L + 2D	404	8–10	100.00	23.70	0.55	280.00	43.09	
	0	10–12	0.00	0.00	46.00	0.00	-	
	854	0–8	100.00	474.00	0.55	280.00	861.82	27.91
10 L + 4	404	8–10	100.00	23.70	55.00	280.00	0.43	
	0	10–12	0.00	0.00	55.00	0.00	-	
	854	0–8	100.00	474.00	0.55	280.00	861.82	30.43
12 L	404	8–10	100.00	23.70	0.55	280.00	43.09	
	404	10–12	100.00	23.70	0.55	280.00	43.09	
	854	0–8	100.00	474.00	0.55	280.00	861.82	31.22
12 L + 4	404	8–10	100.00	23.70	0.55	280.00	43.09	
	404	10–12	100.00	23.70	55.00	280.00	0.43	
	0	12–14	0.00	0.00	55.00	0.00	-	

### Measurements

Photosynthetically active radiation (PAR) and the PAR of blue (460 nm), red (660 nm) and white (5700 K) light was measured using a LightScout Quantum Meter and Far Red (735 nm) was measured using a LightScout Red/Far Red Meter (spec-meters.com)/ (accessed on October 19, 2023).

Flowering development was assessed on a weekly basis starting from Day After Cloning 37 (DAC 37) to ascertain the presence or absence of pistils (scored as 1/0) and trichomes (scored as 1/0). Plant height was measured weekly, commencing from DAC 30.

The harvest took place on DAC 70. Plants were excised at the base, and then the whole plant was weighed (whole plant FW). The large fan leaves were removed, and the flowers were manually stripped from the stem and trimmed using a mechanical trimmer (TrimPro ROTOR, Canada). The trimmed flowers were re-weighed (trimmed flower fresh weight) and placed into a foil tray. The flowers were dried in a dedicated drying room at 21 °C and 50% humidity until no further reduction in weight was observed (9 days). The samples were then re-weighed, and the total flower dry weight (g plant^− 1^) was calculated.

### Analytics

The methodology for quantifying cannabinoids has been previously documented^28^. For each of the 6 treatments and control groups, 4 biological replicates were analyzed for THCA and THC (in the cases of Hindu Kush and Northern Lights) and CBDA and CBD (for Cannatonic), encompassing three different genotypes. Total THC and CBD was then calculated with the following formulae: Total THC = THC + (THCA*0.877) and Total CBD = CBD + (CBDA*0.877).

From each individual plant, three florets were randomly selected from the dried subsample of flower material and finely ground into a powder using liquid nitrogen. A 0.1-gram sub-sample was employed for cannabinoid quantification, which involved sonication in 100% ethanol using a SONICLEAN instrument (Soniclean^®^, Dudley Park, Australia) operating at 50/60 Hz for 30 min, followed by centrifugation at 10,000 rpm for 10 min. The resulting ethanolic extracts were preserved at -10 °C until needed. Subsequently, these extracts were diluted and subjected to analysis through high-performance liquid chromatography coupled with quadrupole time-of-flight mass spectrometry (UHPLC-QToFMS, Agilent Technologies, Santa Clara, CA, USA). Separation was achieved using a reversed-phase column (Agilent Infinity Poroshell 120, HPH-C18, 2.1 × 150 mm, 2.7 μm, narrow bore LC column, Agilent Technologies, Santa Clara, CA, USA) with mobile phases consisting of methanol-water-acetonitrile and acetonitrile, both containing 0.1% formic acid (v/v). The analysis was executed using Quant Analysis Software 10.2 (Agilent Technologies, Santa Clara, CA, USA), and cannabinoid peaks were identified based on their mass-to-charge (m/z) values and retention times by calibration against cannabinoid standards (Novachem, VIC, Australia).

The total yield of each cannabinoid in g per plant (g plant^− 1^) was calculated as follows:

((%Cannabinoid/100) x Total flower dry weight in grams) = g cannabinoid plant^− 1^.

### Power use calculations

The kWh used by each of the treatments was calculated using an amp meter (Hioki AC Clamp Meter 3280–10 F, Hioki, Singapore), with a resolution of 0.01 A. The lights were LEDs so there was no “ramp up” or down period and so the amperage requirement for a single setting (e.g. 12 L–2 h of FR in the dark) does not change. At a single timepoint, the amp meter was fitted to each light treatment and the current draw (power drawn from the grid) was recorded. Amps were then converted to kW and multiplied by the photoperiod duration (e.g. 12 h at full spectrum + 2 h of far-red). The carbon emissions per kWh for New South Wales (NSW) Australia were sourced from The Australian Greenhouse Accounts Factors 2022^29^.

### Statistical analyses

All graphics and statistical analyses were performed in R 3.1^43^. One way ANOVA’s were performed per variety and Tukey HSD tests were used to identify pair-wise differences between treatments significance for all tests was set at ≤ 0.05. In circumstances were the ANOVA returned a significant statistic but the Tukey HSD test could not identify difference at the ≤ 0.05, a Least Significant Differences (LSD) test was performed using the agricolae package in R^43,44^.

## Data Availability

Data are contained within the article and available from the corresponding author upon request.
